# Effect of Intolerance of Uncertainty on Perceived Cognitive Function Among Breast Cancer Patients Before Chemotherapy

**DOI:** 10.3390/cancers17111884

**Published:** 2025-06-04

**Authors:** Yesol Yang, Alai Tan, Sagar D. Sardesai, Nicole O. Williams, Margaret Gatti-Mays, Daniel G. Stover, Preeti K. Sudheendra, Robert Wesolowski, Stephanie M. Gorka, Leah M. Pyter

**Affiliations:** 1 College of Nursing, The Ohio State University, Columbus, OH 43210, USA; yang.6310@osu.edu (Y.Y.); tan.739@osu.edu (A.T.); 2 Cancer Control Program, The Ohio State University-James: Cancer Treatment and Research Center, Columbus, OH 43210, USA; 3 Division of Medical Oncology, Deparment of Internal Medicine, The Ohio State University, Columbus, OH 43210, USA; sardesai@osumc.edu (S.D.S.); nicole.williams@osumc.edu (N.O.W.); margaret.gatti-mays@osumc.edu (M.G.-M.); daniel.stover@osumc.edu (D.G.S.); preeti.sudheendra@osumc.edu (P.K.S.); robert.wesolowski@osumc.edu (R.W.); 4 Department of Psychiatry and Behavioral Health, The Ohio State University Wexner Medical Center, Columbus, OH 43210, USA; stephanie.gorka@osumc.edu; 5 Institute for Behavioral Medicine Research, The Ohio State University, Columbus, OH 43210, USA

**Keywords:** breast cancer, cognitive impairment, intolerance of uncertainty, anxiety

## Abstract

**Background**: Cancer-related cognitive impairment (CRCI) is one of the most frequently reported symptoms by breast cancer patients. However, it remains unclear precisely what contributing factors are present among chemotherapy-naïve breast cancer patients that contribute to CRCI. Thus, it is essential to identify potential factors related to CRCI that may occur before chemotherapy so that interventions can be employed to help prevent the worsening of CRCI. **Objective**: This study examined the association between intolerance of uncertainty (IU) and cognitive function among breast cancer patients before chemotherapy and explored whether anxiety mediates this association. **Methods**: A total of 58 females diagnosed with stage I-III breast cancer and scheduled for chemotherapy were included in this study. Data on cognitive function, IU, anxiety, and other relevant information were analyzed. **Results**: We found that higher IU was associated with higher anxiety and such higher anxiety was subsequently associated with lower cognitive function being reported by breast cancer patients who were scheduled for chemotherapy. The association between IU and cognitive function was largely mediated through anxiety with standardized *β* = −0.19 (SE = 0.07) for the indirect association via anxiety and *β* = −0.2 (SE = 0.12) for the total association. **Conclusions**: IU shows an impact on cognitive function. Therefore, it is necessary to assess IU before chemotherapy, which may help detect patient risk for cognitive impairment early.

## Simple Summary

Cancer-related cognitive impairment (CRCI) is one of the symptoms that breast cancer patients frequently experience, even before chemotherapy. Despite its high prevalence, it is unclear what factors contribute to CRCI among chemotherapy-naïve breast cancer patients. Several studies have suggested that individuals with difficulty tolerating uncertainty (i.e., intolerance of uncertainty [IU]) are more likely to experience cognitive problems. Consistent with these findings, our study also indicates that higher IU is related to higher anxiety and such higher anxiety is linked to more cognitive problems. This result suggests that health care providers need to screen breast cancer patients with high IU and triage those at risk for CRCI.

## 1. Introduction

Cancer-related cognitive impairment (CRCI) is one of the most frequently reported symptoms by breast cancer patients [1]. Early CRCI studies focused on chemotherapy as the primary cause of CRCI and the condition was often referred to as “chemo brain” or “chemo fog” [1]. However, a growing body of literature theorizes that CRCI is attributable to a combination of cancer, chemotherapy, and associated distress [1,2]. Studies revealed that approximately 30–40% of breast cancer patients experience CRCI before chemotherapy [3,4,5,6]. Investigators further suggested that factors such as psycho-social components, comorbidities, or aging can contribute to the incidence of CRCI before chemotherapy [3,7,8]. However, it remains unclear precisely what contributing factors are present among chemotherapy-naïve breast cancer patients that contribute to CRCI. Thus, it is essential to identify potential factors related to CRCI that may occur before chemotherapy so that interventions can be employed to help prevent the worsening of CRCI.

Research has shown that individual differences in tolerating uncertain/unpredictable futures (i.e., intolerance of uncertainty [IU]) contribute to developing anxiety and other forms of psychopathology [8,9,10]. IU is an individual difference factor that underlies how individuals respond to stressors and threats [11,12]. Uncertainty is universally aversive, but individuals high in IU are more likely to interpret uncertainty as distressing or threatening and show maladaptive cognitive and behavioral responses when facing uncertain situations [11,13,14,15]. In alignment with these studies, Hebert and Dugas (2019) have depicted in the IU model that those who have high IU are more likely to notice uncertain situational triggers (e.g., health outcomes), experience uncertainty (i.e., an internal state of not knowing), and make negative interpretations of uncertainty; thus, they are more likely to develop anxiety symptoms [16]. In that model, IU is described as a core construct for developing anxiety symptoms.

Emerging evidence indicates that IU is a key transdiagnostic mechanism for affective symptoms, including anxiety, depression, or worry [8,9,10]. Furthermore, several non-cancer studies have shown that elevated IU is related to impaired cognitive function, including decision-making, problem-solving, and attentional processes [17,18]. The relationship between IU and cognitive–affective symptoms identified in the non-cancer population supports the necessity of the current study, which investigates the effect of IU on cognition in breast cancer patients, particularly in those who have not yet undergone chemotherapy. Given that anxiety is a known contributor to CRCI [19,20,21], it is plausible that breast cancer patients high in IU may be particularly prone to experience anxiety symptoms from the initial diagnostic process to throughout cancer treatments, resulting in lower cognitive function. In support of this hypothesis, cancer studies have also shown that those with high IU tend to experience higher levels of affective symptoms, including anxiety, depression, worry, stress, fear, or psychological distress [22,23,24,25,26,27,28,29,30]. Similarly, our recent study on breast cancer survivors 1–4 years post-chemotherapy showed that higher self-reported IU is related to higher anxiety, which in turn leads to poorer perceived cognitive function [31].

Based on our work and that of others’, it is plausible that the relationship between IU and cognitive function exists pre-chemotherapy, though to the best of our knowledge, this relationship has not been examined in the context of breast cancer. The present study therefore examined the association between self-reported IU and perceived cognitive function among breast cancer patients prior to chemotherapy and then explored the mediation effect of anxiety on this association. Gaining a better understanding of IU’s role will be the basis for developing a mechanistic model for CRCI to aid in the development of prevention and intervention strategies.

## 2. Materials and Methods

### 2.1. Design and Sample

This was a cross-sectional analysis of data collected for breast cancer patients before chemotherapy. Recruitment for the parent study [32] was conducted at the Ohio State University Stefanie Spielman Comprehensive Breast Center in Columbus, Ohio, USA. Eligibility criteria for breast cancer patients in the parent study were as follows: (1) female at birth; (2) recently diagnosed with stage IA-IIIB breast cancer; and (3) had a plan to be treated with anti-neoplastic chemotherapy. Individuals who were older than 80 years old, treated with chemotherapy and/or radiation, and had a history of prior malignancy, as well as a diagnosis of cognitive impairment through a medical chart review, were deemed ineligible for participation in the parent study. A total of 58 patients who completed data on IU, anxiety, and cognition before chemotherapy were included in this analysis.

### 2.2. Data Collection

Prior to data collection, this research received approval from the institutional review board of The Ohio State University and all participants provided informed consent. At the breast cancer outpatient clinic, individuals were initially screened using medical records to confirm their eligibility based on the criteria. Subsequently, researchers met with potentially eligible participants, explained the purpose and procedure of the study, and then obtained written consent forms from those willing to participate. Participants completed assessments via REDCap (https://project-redcap.org/) or at home on paper, and those assessments included PROMIS cognitive function and anxiety questionnaires and self-report measures of IU.

### 2.3. Instruments

Subjective cognition: Patient-Reported Outcomes Measurement Information System (PROMIS) Cognitive Function questionnaire (v2.0—Abilities Subset—Short Form 8a) was used to assess perceived cognitive function, which has been validated in breast cancer patients (Cronbach’s α = 0.98) [33,34]. This measure is a metric standardized to the T-score (mean = 50; SD = 10), and a score of 50 is the average for the United States general population [33,34]. A higher score indicates better cognitive function.

Anxiety: Self-reported anxiety was assessed using the Patient-Reported Outcomes Measurement Information System (PROMIS) Bank v1.0 (Emotional Distress—Anxiety—Short Form 8a) [35]. This instrument measures the severity of anxiety over the past 7 days using a five-point Likert-type scale, which has demonstrated high validity and reliability in breast cancer patients (α = 0.94) [34]. This measure also follows the T-score metric (mean = 50, SD = 10), with higher scores indicating higher levels of anxiety symptoms.

Intolerance of uncertainty: The 12 items of the Intolerance of Uncertainty Scale (IUS-12) were used to measure individuals’ intolerance of uncertainty [36]. The items on this measure are rated on a five-point Likert-type scale, from 1 (“Not at all characteristic of me”) to 5 (“entirely characteristic of me” 5). This measure has demonstrated good validity and reliability in cancer patients (α = 0.82–0.90) [28,29]. The IUS-12 ranges from 12 to 60, with higher scores indicating greater IU.

### 2.4. Statistical Analysis

Descriptive statistics were used to summarize sample characteristics and their level of IU, anxiety, and self-reported function before chemotherapy. Pearson correlation coefficients were used to examine the pair-wise associations among IU, anxiety, and cognitive function. We conducted path analysis to explore the mediation role of anxiety (mediator) in the association between IU (predictor) and cognitive function (outcome). The percentile bootstrapping approach with 1000 samples was used to estimate the indirect (or mediation) effects of IU on cognitive function via anxiety. We reported both unstandardized and standardized estimates from the path models. R package “PermCor” 4.2.2 version was used to derive robust permutation-based *p*-values for Pearson correlation coefficients [37,38]. All other analyses were conducted using SAS 9.4 (SAS institute, Cary, North Carolina).

### 2.5. Power Analysis

We conducted post hoc power analysis for this secondary data analysis. Under a fixed sample size of n = 58, the study has ≥80% power to detect a Pearson correlation of *r* ≥ 0.36 with a two-sided α of 0.05. Using the MedPower program by Kenny [39] and a two-sided α = 0.05, the sample size has 80% power to detect the indirect effect between IU and cognitive function with estimated standardized coefficients of 0.47 for the path of IU to anxiety and −0.40 for the path of anxiety to cognitive function, given a standardized coefficient of −0.08 for the direct path of IU to cognitive function.

## 3. Results

### 3.1. Sample Characteristics

Fifty-eight breast cancer patients had a mean age of 48.2 years (SD = 10.2). Of those 58 patients, approximately 33% were between 50 and 59, followed by 40–49 years (29.3%), less than 40 years (25.9%), and over 60 years (12.1%). The majority populations were non-Hispanic (96.6%) and White (91.4%), currently married (74.1%), and had at least secondary education (53.4%). More than half of them were employed full time (67.2%), pre-menopausal (60.3%), and diagnosed with stage I breast cancer (51.7%). Detailed demographic and clinical information of participating breast cancer patients is summarized in Table 1.

### 3.2. Correlation Analysis Between IU, Anxiety, and Self-Reported Cognitive Function

The sample had an average self-reported cognitive function score of 48.5 (SD = 10.0), IU score of 23.6 (SD = 8.0), and anxiety score of 51.4 (SD = 9.2). Their pair-wise correlations are presented in Table 2. A higher IU was significantly associated with a higher level of anxiety (r = 0.47, *p* = 0.002), and higher anxiety was associated with lower cognitive function (r = −0.39, *p* = 0.009). Additionally, higher IU was marginally associated with lower cognitive function (r = −0.26, *p* = 0.049).

### 3.3. Effects of IU on Cognitive Function

**Figure 1:**
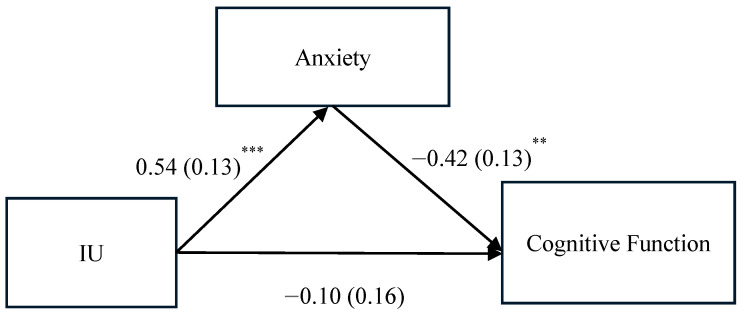
Path model results. Unstandardized path coefficients (SE) were presented. ** *p* < 0.01, *** *p* < 0.001. The corresponding standardized estimates were 0.47 (0.10) for the IU to anxiety path, −0.40 (0.12) for the path of anxiety to cognitive function, and −0.08 (0.13) for the path of IU to cognitive function.

As noted in the path model with unstandardized estimates (Figure 1), higher IU was associated with higher anxiety (*β* = 0.54, *p* < 0.001) and, subsequently, higher anxiety was associated with lower cognitive function (*β* = −0.42, *p* < 0.01). The path model indicates that anxiety mediates the association between IU and cognitive function. Also from the path model, we further partitioned the total association between IU and cognitive function into direct and indirect associations (see Table 3). The results suggest that about two-thirds of the total association between IU and cognitive function was mediated via anxiety, while only one third was attributable to the direct association.

## 4. Discussion

To our knowledge, this is the first study that investigates the association between self-reported IU and perceived cognition among pre-chemotherapy breast cancer patients. In this study, the mean score of cognitive function was relatively lower, but anxiety was relatively higher than the normal population [4,33,34,35]. Previous studies have supported our findings, showing that cognitive decline can be found even before the initiation of cancer treatments due to multiple factors related to cancer diagnosis, such as psychological stress or biological disruption [4,5]. Also, we found that the average IU score was lower than the cutoff of 28 to distinguish individuals with clinical general anxiety disorders from nonclinical cases [40].

Consistent with our hypothesis, we found that greater IU is related to higher anxiety and, subsequently, such higher anxiety is related to lower cognitive function of chemotherapy-naïve breast cancer patients. In other words, anxiety mediates the association between IU and cognitive function. The indirect association between IU and cognitive function via anxiety can be attributed to two-thirds of the total association between IU and cognitive function.

Our findings on the indirect effect of IU on cognitive function are aligned with our previous study conducted on post-menopausal breast cancer survivors 1–4 years post-chemotherapy [31]. Our past research found that breast cancer survivors who were high in IU showed an increased level of anxiety symptoms, and such higher anxiety was related to their low cognitive function. This past result, combined with the current study, supports the idea that IU could be a root cause of cancer-related anxiety and subsequent CRCI. Future studies are needed to examine the causal relationship between IU and cognitive function; this understanding will help researchers develop a risk prediction model for CRCI that can be applied to breast cancer patients at the initial stage of the cancer journey.

In contrast to our study hypothesis, we did not find a direct effect of IU on perceived cognitive function, which is aligned with our past study [31]. One possible explanation for this current finding is that chemotherapy-naïve patients included in this study maintained overall good cognitive function because they were relatively young and early-stage cancer patients, thus failing to show an association with IU. Another potential explanation is that the subjective assessment used in both studies (current and past [31]) to measure cognitive function may tap into other symptoms (e.g., anxiety) [41]. For this reason, IU may not show a direct relationship with cognitive function in both studies [31]. Lastly, the self-report of IU has excellent psychometric properties, but its usefulness can be compromised if study participants misunderstand the target construct. IU can also be assessed objectively using a no-predictable-unpredictable (NPU) threat task [42]. This task involves exposing individuals to uncertain threats/situations (i.e., uncertainty-inducing) and measuring their objective behavioral (i.e., eyeblink) and neural reactivity (i.e., brain activity) during uncertain threats [42]. Future studies need to include multimodal assessments of IU (subjective and objective measures of IU) to better examine the link between IU and cognitive function.

Many studies have shown that IU amplifies negative affect (e.g., anxiety) and promotes maladaptive coping (e.g., alcohol use) [43,44,45]. Recent studies showed that interventions targeting IU led to measurable reductions in subjective IU and symptoms of anxiety in adults [9,46,47,48,49], suggesting that IU is a modifiable construct. Furthermore, a recent meta-analysis reported that IU-focused interventions lead to a much greater reduction in anxiety symptoms than interventions focused on anxiety symptoms [50]. These findings are promising and support the development of multimodal interventions for CRCI. Multimodal interventions, which consist of combining various interventions to target different mechanisms underlying CRCI, have been considered ideal for maintaining or improving cancer patients’ cognition, but they are under-researched [51]. The current study findings provide the foundation for the development of multimodal strategies that may help treat CRCI. For example, for breast cancer patients with CRCI, interventions that combine IU-focused interventions and cognitive rehabilitation could be provided, and such multimodal interventions are expected to be more effective in improving CRCI [41,52]. More research is needed to confirm whether IU is a modifiable construct in the context of breast cancer and whether IU-focused intervention changes cancer-related symptoms, including anxiety and CRCI.

Moreover, IU has been proposed as a trait-like disposition that remains stable over time [42,53,54,55,56], whereas others showed that IU can change over time [10,46,56]. Based on our current and previous studies, although there are some age differences in breast cancer patients involved between the two studies, IU measured at post-chemotherapy (past study [31]) was relatively higher than that assessed at pre-chemotherapy (current study). These findings suggest the possibility of individual variability in IU over time. Thus, future studies are needed which examine IU longitudinally and determine if the changes in IU correspond to changes in cognitive function in breast cancer patients. Once the longitudinal relationship between IU and cognitive function is identified, this knowledge will support that IU can be an essential target to prevent and intervene in anxiety and, furthermore, CRCI.

### 4.1. Strengths and Limitations

This study has strengths, including the participation of a chemotherapy-naïve breast cancer cohort with diverse age groups. To our knowledge, it is the first original study that examined the association between self-reported IU and the perceived cognitive function of breast cancer patients. We acknowledge that this study has several limitations. First, we used data obtained at one time point, prior to chemotherapy; thus, we were unable to establish a causal relationship between IU and cognitive function. It is advised that mediation analysis using cross-sectional data should only be considered when there is a solid theoretical basis and implicated timing by nature of the constructs [57]. Our hypothesized mediation model is based on the theoretical framework by Hebert and Dugas [16] that suggests that IU leads to emotional, cognitive, and behavioral sequelae. IU has been indeed evidenced as a ubiquitous dispositional characteristic and risk factor for anxiety [8]. Thus, it is reasonable to assume IU precedes anxiety even in cross-sectional data. Second, the study participants were predominantly in the early stage of breast cancer and were non-Hispanic White females, which could result in limited generalizability and clinical utility of the study results. Our sample size was relatively small but had sufficient power for the estimated indirect association between IU and cognitive association via anxiety based on a post hoc power analysis. Given the small sample size, we did not proceed with further covariate adjustment. Therefore, we caution against drawing definite conclusions from the study. Instead, the study findings serve as proof-of-concept and warrant future large-scale longitudinal studies to examine the mechanism pathway of the link between IU and cognition. Lastly, this study only used a self-reported measure of IU. Although this self-report measure exhibits excellent psychometric properties, it may not fully capture the multifaceted nature of IU. Thus, future studies need to include objective behavioral and neural measures and examine the IU construct.

### 4.2. Clinical Implications

Our findings suggest that IU drives anxiety and leads to CRCI. Thus, assessing IU before chemotherapy could be key to detecting the risk for CRCI and related psychological symptoms (e.g., anxiety) before they develop during the course of cancer. Health care providers would be optimally poised to screen breast cancer patients high in IU and triage those at risk for CRCI. Furthermore, interventions that promote building supportive relationships with others could regulate IU and consequently benefit CRCI. Studies have shown that individuals who receive greater social support and build positive social relationships better cope with uncertainty, leading to a decrease in anticipatory anxiety [58]. In contrast, without social support, those with high IU remain chronically anxious, resulting in negative cognitive outcomes [59,60]. Thus, as clinicians, it is important to educate patients about the positive effects of social support and encourage them to participate in social activities.

## 5. Conclusions

In summary, we found that chemotherapy-naïve breast cancer patients who had higher IU showed higher anxiety and that this anxiety was related to lower perceived cognitive function. This finding suggests that IU could be a main contributor to anxiety, which leads to CRCI. Thus, assessing IU before chemotherapy could be a potential strategy to prevent and/or treat CRCI in breast cancer patients who are at risk. Additional research is warranted to investigate whether IU can serve as a predictor for CRCI. Gaining such an understanding will ultimately contribute to developing a risk prediction model for CRCI and will facilitate targeted prevention and treatment strategies for CRCI.

## Figures and Tables

**Table 1:** Sample characteristics.

Characteristics	N (%)
**Age, years (mean = 48.2, SD = 10.2)**	
<40	15 (25.9)
40–49	17 (29.3)
50–59	19 (32.8)
60+	7 (12.1)
**Ethnicity**	
Hispanic	2 (3.4)
Not Hispanic	56 (96.6)
**Race**	
White	53 (91.4)
Other	5 (8.6)
**Currently Married**	
Yes	43 (74.1)
No	15 (25.9)
**Education**	
<BA	27 (46.6)
BA	17 (29.3)
>BA	14 (24.1)
**Employed full time**	
Yes	39 (67.2)
No	19 (32.8)
**Postmenopausal**	
Yes	23 (39.7)
No	35 (60.3)
**Breast Cancer Stage**	
I	30 (51.7)
II	26 (44.8)
III	2 (3.4)

**Table 2:** Correlation among IU, anxiety, and cognitive function.

	N	Mean (SD)	Pearson Correlation, *r*	
			Anxiety	Cognitive Function
IU	58	23.6 (8.0)	0.47 (*p* = 0.002)	−0.26 (*p* = 0.049)
Anxiety	58	51.4 (9.2)	-	−0.39 (*p* =0.009)
Cognitive function	58	48.5 (10.0)	-	-

**Table 3:** Effects of IU on cognitive function.

	Effect	*β* (*SE*)	*p*-Value
**Unstandardized Estimate**	Total	−0.33 (0.15)	0.030
	Direct	−0.10 (0.16)	0.539
	Indirect (via anxiety)	−0.23 (0.09)	0.011
**Standardized Estimate**	Total	−0.27 (0.12)	0.025
	Direct	−0.08 (0.13)	0.539
	Indirect (via anxiety)	−0.19 (0.07)	0.007

## Data Availability

Data will be accessible upon reasonable request to the corresponding author.
